# Polymorphisms in the *RNASE3* Gene Are Associated with Susceptibility to Cerebral Malaria in Ghanaian Children

**DOI:** 10.1371/journal.pone.0029465

**Published:** 2011-12-27

**Authors:** Bright Adu, Daniel Dodoo, Selorme Adukpo, Ben A. Gyan, Paula L. Hedley, Bamenla Goka, George O. Adjei, Severin O. Larsen, Michael Christiansen, Michael Theisen

**Affiliations:** 1 Department of Clinical Biochemistry and Immunology, Statens Serum Institut, Copenhagen, Denmark; 2 Noguchi Memorial Institute for Medical Research, University of Ghana, Accra, Ghana; 3 Department of Biomedical Sciences, University of Stellenbosch, Cape Town, South Africa; 4 Department of Child Health, University of Ghana Medical School, College of Health Sciences, University of Ghana, Accra, Ghana; 5 Centre for Tropical Clinical Pharmacology and Therapeutics, University of Ghana Medical School, College of Health Sciences-University of Ghana, Accra, Ghana; National de la Santé et de la Recherche Médicale - Institut Cochin, France

## Abstract

**Background:**

Cerebral malaria (CM) is the most severe outcome of Plasmodium falciparum infection and a major cause of death in children from 2 to 4 years of age. A hospital based study in Ghana showed that P. falciparum induces eosinophilia and found a significantly higher serum level of eosinophil cationic protein (ECP) in CM patients than in uncomplicated malaria (UM) and severe malaria anemia (SA) patients. Single nucleotide polymorphisms (SNPs) have been described in the ECP encoding-gene (RNASE3) of which the c.371G>C polymorphism (rs2073342) results in an arginine to threonine amino acid substitution p.R124T in the polypeptide and abolishes the cytotoxicity of ECP. The present study aimed to investigate the potential association between polymorphisms in RNASE3 and CM.

**Methodology/Principal Findings:**

The RNASE3 gene and flanking regions were sequenced in 206 Ghanaian children enrolled in a hospital based malaria study. An association study was carried out to assess the significance of five SNPs in CM (n = 45) and SA (n = 56) cases, respectively. The two severe case groups (CM and SA) were compared with the non-severe control group comprising children suffering from UM (n = 105). The 371G allele was significantly associated with CM (p = 0.00945, OR = 2.29, 95% CI = 1.22–4.32) but not with SA. Linkage disequilibrium analysis demonstrated significant linkage between three SNPs and the haplotype combination 371G/*16G/*94A was strongly associated with susceptibility to CM (p  = 0.000913, OR = 4.14, 95% CI = 1.79–9.56), thus, defining a risk haplotype. The RNASE3 371GG genotype was found to be under frequency-dependent selection.

**Conclusions/Significance:**

The 371G allele of RNASE3 is associated with susceptibility to CM and forms part of a risk associated haplotype GGA defined by the markers: rs2073342 (G-allele), rs2233860 (G-allele) and rs8019343 (A-allele) respectively. Collectively, these results suggest a hitherto unrecognized role for eosinophils in CM pathogenesis.

## Introduction

Cerebral malaria (CM) is the most severe outcome of *Plasmodium falciparum* infection and a major cause of death in children particularly from ages 2 to 4 years living in malaria endemic areas and accounts for about 80% of fatal malaria cases [1]. To date, the exact mechanism underlying the pathogenesis of CM remains largely speculative. However, the observation that only 8 to 20% of the 225 million annual malaria cases worldwide develop into CM [2]–[3] suggests that, CM is a sub-population-specific targeted syndrome. Several factors have been implicated in the development of CM with both host and parasite genetics considered major contributors. None of the two leading hypotheses proposed to explain CM pathogenesis; the sequestration [4] and the inflammation hypotheses [5] suggests a possible role for eosinophils and/or their secretory products. On the other hand, several cells such as red blood cells (RBCs), platelets, lymphocytes, neutrophils and monocytes, their ligands and receptors have all been considered potential contributors to CM pathogenesis mainly through their participation in microvascular occlusion [6]. Consequently, the greater number of CM immunogenetic studies have focused on these cells and polymorphisms of endothelial cell surface receptors known to interact with parasite factors on infected RBCs (iRBCs) and the cytokines modulating the expression of these adhesion molecules [6]. Indeed, polymorphisms in some of these molecules, such as tumour necrosis factor (TNF)-α [7]–[9], interleukin (IL)-1β [10]–[11], intercellular adhesion molecule (ICAM)-1 [10], [12], complement receptor (CR)-1 [10], IFN-γ receptor 1 (IFNGR1) [13], and inducible (iNOS), neuronal (nNOS) and endothelial (eNOS) nitric oxide synthase [14]–[16] have been quite extensively studied for their association with CM but often with either no association found or contradictory results. Eosinophils have granules which contain four very basic, cytotoxic proteins; eosinophil cationic protein (ECP), eosinophil peroxidase (EPO), eosinophil-derived neurotoxin/eosinophil protein X (EDN/EPX) and major basic protein (MBP) [17]–[18]. Both ECP and EDN have neurotoxic properties with ECP being the more potent of the two [18]–[19]. A hospital based study in Ghana involving CM patients showed that *P. falciparum* infection induces eosinophilia and also found a significantly higher level of ECP in CM patients than in uncomplicated malaria (UM) and severe malarial anemia (SA) patients [20]. Furthermore, an *in vitro* study has demonstrated that ECP could inhibit *P. falciparum* growth in a dose-dependent manner [21]. Thus, ECP might be important in the control of *P. falciparum* infection but may also play a role in CM pathogenesis. Here, we report the results of a hospital based malaria study with population genetic data which supports the role of frequency dependent selection genes involved in resistance or susceptibility to infectious disease.

Single nucleotide polymorphisms (SNPs) have been described in ECP (NP_002926.2), encoded by *RNASE3* (NM_002935.2), which alter both ECP serum levels [22] and function [23]. More precisely, it has been shown that the c.371G>C polymorphism (rs2073342) which results in an arginine to threonine amino acid substitution (p.R124T) in the polypeptide and abolishes the cytotoxicity of ECP and the G- and C-alleles have been associated with allergic asthma [24] and helminth infections [25] respectively. In addition, the 371C-allele was associated with non-allergic asthma in a family-based study involving individuals from Norway and the Netherlands [26]. Interestingly, in a more recent publication [27], Jönsson and colleagues found an association with the 371GG genotype and non-allergic asthma in a cohort of Swedes and Estonians, contradicting their previous findings [24]. However, this paradox may have been due to differences in the methods used in characterizing patients in the two studies. Also, the G-allele of the 3′UTR c.*16G>C SNP, (rs2233860), correlated with higher intracellular ECP [24] and was associated with allergic rhinitis in Koreans [28]. Furthermore, a study in India also found an association of the T-allele of the 3′UTR c.*94A>T, SNP (rs8019343) with helminth infection in one patient [29]. In the present study, we have sequenced *RNASE3* and investigated the association between SNPs and susceptibility to CM in a hospital based malaria study involving CM, SA and UM patients. The population distribution of the CM associated -allele, -genotype and -haplotype identified were also compared with that of a non-malaria population (Danes) to investigate the extent of possible selection due to CM. To our knowledge, this is the first study in which polymorphisms in *RNASE3* have been associated with susceptibility to CM.

## Methods

### Ethics Statement

This study is part of a hospital based malaria study conducted at Department of Child Health, Korle-Bu Teaching Hospital, Ghana and the Noguchi Memorial Institute for Medical Research, Ghana between 2002 and 2004. Ethical approval was given by the Institutional Review Board of the Noguchi Memorial Institute for Medical Research (NMIMR) and the Ethical Committee of the University of Ghana Medical School, Korle-Bu, Ghana. Written informed consent was given by the parents and guardians of children before they were enrolled into the study. Ethical approval for Danish blood donor samples was given by the Scientific Ethics Committee of Copenhagen and Frederiksberg, Denmark.

### Malaria patients

The study enrolled children from 0.5 to 13 years of age visiting the Child Health Department of the Korle-Bu Teaching Hospital (KBTH), Accra, for medical care during the study period. The KBTH is the major referral hospital serving the southern part of Ghana originally inhabited largely by the Akan and Ga-Adangbe ethnic groups. A study involving individuals from the same study area found no significant population substructure between the Akan and Ga-Adangbe ethnic groups using data from 372 autosomal microsatellite loci [30]. The clinical and demographic characteristics of the patients were as described elsewhere [31], with some modifications (Table 1). Briefly, all patients had to be febrile (>37.5°C measured within 24 hours of admission), sickle-cell negative and slide positive for asexual *Plasmodium falciparum* parasitaemia with at least some other sign of malaria such as vomiting, diarrhoea, or malaise to be included in the study. Altogether, 206 children were enrolled in the study, 45 of which were CM cases, 56 had SA and 105 were UM cases. CM was defined by unarousable coma (Blantyre coma score of <3) [32], with no other attributable cause of cerebral dysfunction; SA was defined by measured haemoglobin (Hb) levels of <5.0 g/dl with no other attributable cause of anaemia and patients were fully conscious. The criteria used to define UM was same as SA but with a measured Hb of >6.0 g/dl. Thus, this group also includes individuals with mild anaemia. The procedure used for determining parasitological and haematological parameters have been described elsewhere [31]. Buffy coats were obtained from blood samples taken from patients and stored at −80°C for DNA purification.

**Table 1:** Clinical and demographic characteristics of patients with severe malaria and uncomplicated malaria.

Disease	No. of Patients	Hb (g/dl)^T^	Age (yr)^T^	Parasite Density (10^3^/µl)^T^
**CM**	45	6.6 (2.5–10.2)^****^	3.0 (0.5–10.0)^**^	41.4 (2.00–1,296)
**SA**	56	4.0 (2.1–4.9)^****^	2.0 (0.5–12.0)^****^	29.8 (0.01–1,411)^*^
**UM**	105	9.35 (6.1–13.0)	4.0 (0.5–13.0)	60.8 (0.137–4,340)

The number of patients in the various disease categories: cerebral malaria (CM), severe malaria anaemia (SA) and uncomplicated malaria (UM) is shown together with haemoglobin (Hb) levels, age stratification and parasite density distribution.

^T^ Median values with minimum and maximum values (in parenthesis). Values that are statistically significant when compared to UM,

****p<0.0001,

**p = 0.0014,

*p = 0.0222.

### Danish donors

In order to allow for inter-population comparison and analysis, the study also used archived DNA samples purified from a panel of anonymous Danish blood donors (n = 203) obtained for control purposes. These 203 individuals, aged 17 to 67 years were of Danish decent inhibiting central Copenhagen.

### RNASE 3 gene sequencing

Genomic DNA was purified from buffy coat samples using the Maxwell®16 system (Promega, Madison, USA). An approximately 1.5-kb fragment encompassing the promoter region and the 3′UTR was amplified using the M13 (in lower case) tagged sense primer (5′- tgtaaaacgacggccagtGCCTGCCAGAGGACAGTTAT-3′) and the antisense primer (5′- caggaaacagctatgaccGGAGGAGTCAGTGGATGGAA-3′) (Taq Copenhagen, Denmark) in a 25.0 µl reaction containing 40 ng genomic DNA, 10 mM of each primer, 1.25 mM of each dNTP, 1 unit of HotStarTaq® DNA polymerase (Biomol, Germany) and the corresponding 10× HotStar reaction buffer. The PCR conditions were 95°C activation for 15 minutes, 35 cycles of 95°C for 30 sec, 63°C for 45 sec, and 72°C for 1.40 s, with a final extension at 72°C for 10 min. The product was then purified using exonuclease 1 and alkaline phosphatase (ExoSap) reaction and sequenced from both ends with M13 primers the internal sense (5′-CAGTTCTCACAGGAGCCACA-3′) and antisense (5′-AGGTGAACTGGAACCACAGG-3′) primers (Taq Copenhagen, Denmark) in a BigDye® Terminator v3.1 (Applied Biosystem, UK) reaction and analysed with the ABI 3730 DNA Analyser (Applied Biosystem, UK). No significant difference was observed when allele frequencies obtained in the hospital based study were compared with those in a longitudinal malaria cohort (n = 640) study of children (aged 1 to 13 years old) in which less than 10% hospitalisation was recorded (Adu *et al.*, unpublished data). In order to verify the genotyping data a second PCR was performed in which an approximately 1.7 kb product encompassing the original primer sites was amplified from 96 randomly selected individuals, using the upstream sense (5′-tgtaaaacgacggccagtTCCTAACTACTATGCCTGCCTT-3′) and the downstream antisense (5′-caggaaacagctatgaccTCAGCTGATTCACTGCAGCTC-3′) primers (Taq Copenhagen, Denmark). Nucleotide sequencing of the resulting amplicons were in 100% agreement with results obtained for these 96 individuals in the original screen.

### Statistical analysis

The clinical and demographic characteristics of the CM and SA groups were compared to that of the UM group using the Mann Whitney test as implemented in GraphPad Prism v.5.00 (GraphPad Software Inc. La Jolla, CA, USA). Similarly, coma score distribution among the c.371G>C genotypes was compared by the Mann Whitney test. Differences were considered to be statistically significant if *p*<0.05. Haploview v. 4.2 (http://www.broadinstitute.org/haploview) [33] was used to estimate deviations of genotype frequencies from Hardy-Weinberg equilibrium (HWE), visualize linkage disequilibrium (LD) patterns with pairwise r^2^ values and define haplotypes. The adjusted *p*-value for statistically significant differences in the HWE estimations was *p<*0.0125 (correction factor = 4) according to the Boferroni correction for multiple comparisons. *RNASE3* allele and haplotype frequencies in CM and UM patients were compared by multiple logistic regression and adjusted for age and gender using S-Plus 2000 (Insightful Corporation, Seattle WA). There was no statistically significant difference in the allele distribution between SA and UM patients, hence the SA group was excluded from haplotype association analysis. All the possible haplotypes present in the population for the three closely linked SNPs were included in the haplotype association studies. Comparison of haplotype distribution between the Danish and Ghanaian populations was by the chi-square (χ^2^) test. DnaSP v. 5.10 (http://www.ub.edu/dnasp/) [34] was used to estimate Tajima's D to study evidence of selection on the c.371G>C SNP in malaria versus non-malaria endemic populations. To estimate the extent of divergence with respect to this polymorphism in malaria endemic versus malaria non-endemic populations, allele frequencies in our study populations were compared to that of four populations namely, the YRI: Yoruba in Ibadan, Nigeria; CEU: (Utah residents with northern and western European ancestry from the Centre d'Etude du Polymorphisme Humain [CEPH]); CHB: Han Chinese in Beijing, China and JPT: Japanese in Tokyo, Japan obtained from the International HapMap Project database (http://hapmap.ncbi.nlm.nih.gov/cgi-perl/snp_details_phase3?name=rs2073342&source=hapmap28_B36&tmpl=snp_details_phase3). Pairwise F_ST_ distances [35] were calculated for all 6 populations using the POPTREE2 software [36].

## Results

### RNASE3 nucleotide sequence variation in Ghanaians

The *RNASE3* gene and flanking regions were amplified from 206 Ghanaian children enrolled in a hospital based malaria study. Nucleotide sequencing identified a total of six SNPs, one of which was non-synonymous (c.371G>C; p.R124T) corresponding to the previously described arginine/threonine polymorphism which abolishes the cytotoxicity of ECP [23]. Of the remaining SNPs, two (c.-5-43C>T, c.-5-38C>A) are located in the intron and two (c.*16C>G, *94A>T), are located in the 3′UTR (Table 2). The last SNP, *295A>G is located downstream the 3′UTR and was excluded from association analysis as was the c.-5-38C>A polymorphism since the -5-38A-allele was only present in a single individual. The remaining SNPs were all in Hardy-Weinberg disequilibrium (Table 2) except the *295A>G (data not shown).

**Table 2:** Allele frequencies and Hardy-Weinberg estimations in the CM and UM study populations.

Marker	Alleles	Location	Amino acid change	Minor allele (frequency)	HW^a^ *p*-value
**rs2233858**	c.-5-43C>T	Intron	-	T (0.031)	8.70×10^−3^
**rs2073342**	c.371G>C	Exon 2	Arg/Thr	G (0.325)	1.00×10^−4^
**rs2233860**	c.*16G>C	3′UTR	-	C (0.262)	4.14×10^−8^
**rs8019343**	c.*94A>T	3′UTR	-	T (0.145)	5.99×10^−7^

a values are statistically significant if *p*<0.0125 (Bonferroni corrected significance threshold).

### Single marker and haplotype association study

**Figure 1:**
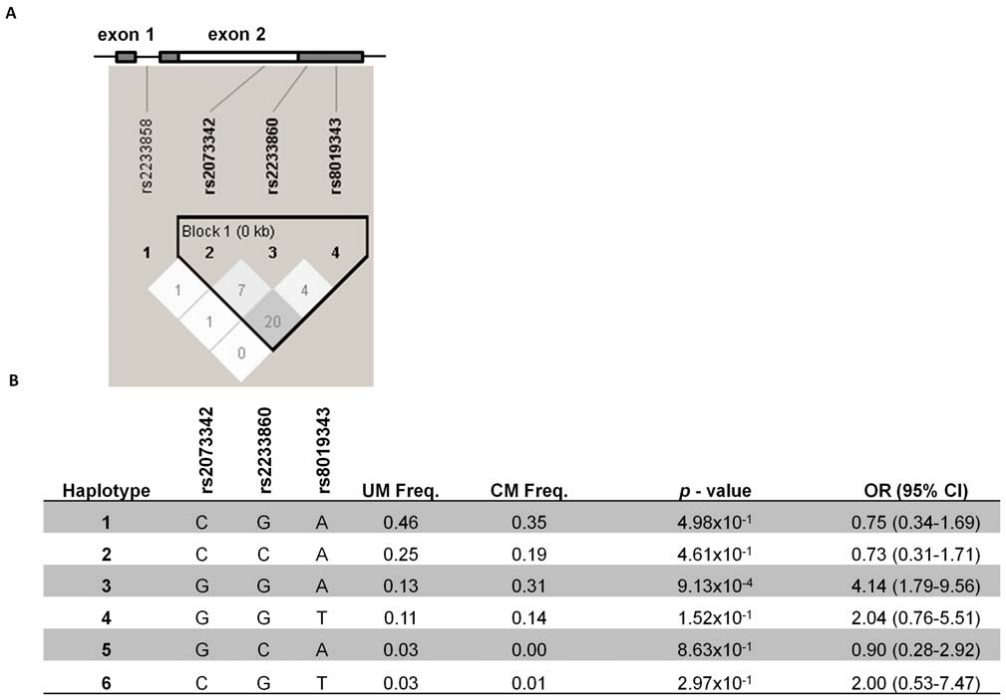
Location of the studied SNPs in *RNASE3*, linkage disequilibrium (LD) patterns and haplotype association analysis. **A**) A shematic of the region of the *RNASE 3* gene (NM_002935.2) and LD plot of the respective SNPs visualised using Haploview v4.2. At the top of the LD plot, the two exons are shown with the intron joining them. Untranslated regions of the exons are indicated by shadings. The LD plot shows pairwise r^2^ values (×100) given in the squares for each comparison between the SNPs. White squares represent r^2^ values equal to 0. Different shades of grey represent r^2^ values between 0 and 1. **B**) Haplotype associations with susceptibility to cerebral malaria (CM) compared to uncomplicated malaria (UM). Odds ratio (OR) and 95% confidence intervals (CI) were determined using multivariate logistic regression controlling for age and gender. The reference groups in the multivariate logistic regression analyses were those without the respective haplotypes. Haplotype 3 (GGA) was significanlty associated with susceptibility to CM.

An association study was carried out to assess the significance of the four SNPs in CM (n = 45) and SA (n = 56) cases, respectively. The two case groups were compared with the non-severe control group comprising children suffering from UM (n = 105) in a hierarchical multivariate logistic regression model adjusting for age and gender. The 371G allele was found to be significantly associated with CM (*p* = 0.00945, OR = 2.29, 95% CI = 1.22–4.32) (Table 3) but not with SA (data not shown). Linkage disequilibrium analysis demonstrated significant linkage between the c.371G>C, c.*16C>G, and c.*94A>T SNPs (Figure 1A), and haplotype analysis was therefore carried out to determine their association with CM (Figure 1B). The haplotype combination 371G/*16G/*94A was strongly associated with susceptibility to CM (*p*  = 0.000913, OR = 4.14, 95% CI = 1.79–9.56) (Figure 1B), thus, defining a risk haplotype (GGA). The other haplotypes showed no significant association with CM.

**Table 3:** Single marker association with cerebral malaria and uncomplicated malaria.

Marker	Alleles	Associated Allele	UM Freq. (n = 105)	CM Freq. (n = 45)	*p* - value	OR (95% CI)
**rs2233858**	C/T	T	0.03	0.04	9.47×10^−1^	1.05 (0.26–4.30)
**rs2073342**	C/G	G	0.27	0.45	9.45×10^−3^	2.29 (1.22–4.32)
**rs2233860**	G/C	G	0.72	0.81	4.94×10^−1^	1.22 (0.68–2.21)
**rs8019343**	A/T	T	0.14	0.16	1.37×10^−1^	0.53 (0.22–1.24)

The frequency of the associated allele in uncomplicated malaria (UM) and CM is shown. Odds Ratios (OR) and 95% confidence intervals (CI) were determined using multivariate logistic regression controlling for age and gender. The reference groups in the multivariate logistic regression analyses were those without the respective alleles.

### Association of c.371G>C genotypes with disease

**Figure 2:**
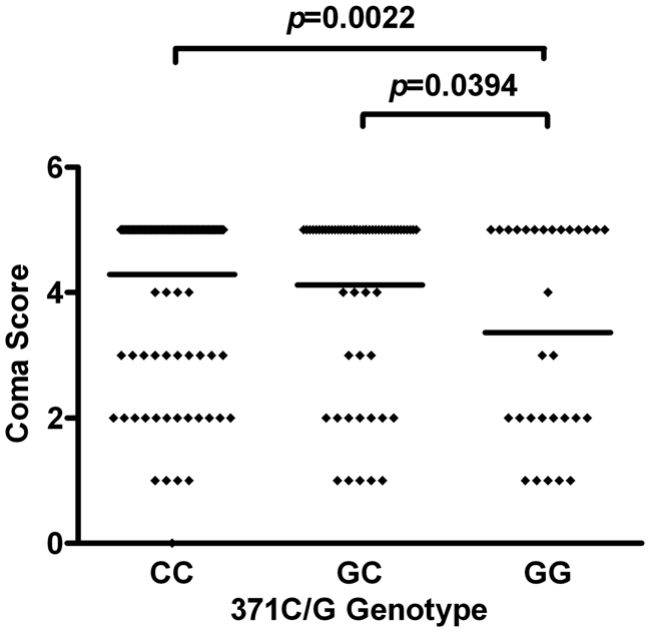
Association of c.371G>C genotypes with coma score in the entire patient population studied. Coma score distribution among individuals with 371 (GG, GC and CC) genotypes is shown. Comparisons that yielded statistical significance are indicated with horizontal lines linking the respective groups at the top of the plots with the *p* – value stated. *P*-values were determined by Mann Whitney test and horizontal lines within plots represent the median of the distribution.

To further assess the importance of the genotype forms of the c.371G>C SNP in CM, the coma score was plotted for each of the three genotypes. The mean coma score in individuals carrying the 371GG genotype was significantly lower as compared to the 371CC (Mann Whitney test, *p* = 0.0022) and the 371CG (Mann Whitney test, *p* = 0.0394) genotypes respectively (Figure 2). On the other hand, levels of parasitaemia did not differ significantly between the three genotypes (data not shown).

### Population differences

**Figure 3:**
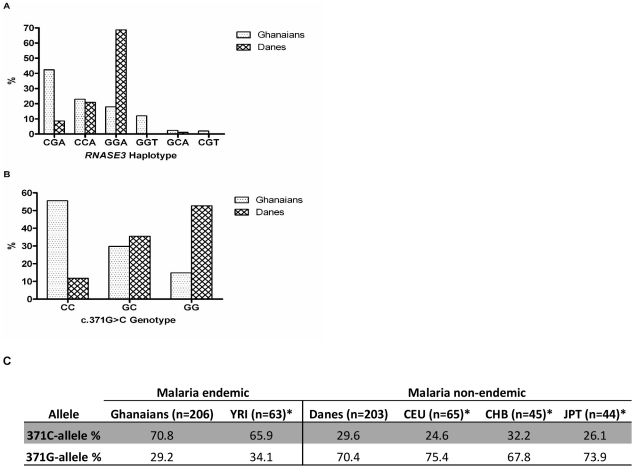
*RNASE3* haplotypes, c.371G>C genotypes and alleles distribution in malaria endemic (Ghanaian) versus non-malaria (Danish) populations. **A**) Distribution of haplotypes defined by the three SNPs (rs2073342, rs2233860 and rs8019343) as from block 1 in Figure 1A in the two populations. **B**) c.371G>C genotype distribution in all Ghanaian subjects compared to Danes. **C**) c.371G>C allelic distribution in the two populations.

The above analyses suggest a strong association between the 371GG genotype and CM. It was therefore of interest to investigate the differences in the genotype distribution of c.371G>C between populations from malaria versus non-malaria endemic regions. *RNASE3* and flanking regions were amplified from Danish blood donors (n = 203). Sequencing analysis identified all the SNPs found in the Ghanaian samples plus a novel SNP (c.214C>T) in exon 2 resulting in an arginine to cysteine polymorphism at position 72. In contrast to the Ghanaian samples, all SNPs in the Danish samples were in Hardy-Weinberg equilibrium (*p*>0.0125). There was a statistically significant difference in the distribution of both the 371GC genotypes (*p*<0.0001, χ^2^-test) and alleles (*p*<0.0001, χ^2^-test) in the Ghanaian and Danish populations (Figure 3B and 3C). The c.371G>C allele distributions were compared to those reported in the International HapMap project database. The 371C allele is dominating in individuals from malaria endemic regions (Ghanaians and YRI) whereas the 371G allele is dominating in individuals from malaria non-endemic populations (CEU, CHB and JPT) (Figure 3C). Pairwise F_ST_ calculations showed a significant genetic divergence between individuals from malaria endemic (Ghanaians and YRI) and malaria non-endemic areas (Danes, CEU, CHB and JPT) (0.2040≤F_ST_≥0.3520) (Table 4). On the other hand, the data showed no significant evidence of population divergence between the Ghanaians and the YRI individuals or among the Danes, CEU, CHB and JPT (Table 4). The distribution of the haplotypes defined by the markers, rs2073342, rs2233860 and rs8019343 were also significantly different (*p* = 0.0010, χ^2^-test for trends) between the populations. Interestingly, the most predominant haplotype in the Danish population was the CM-risk associated haplotype seen in the Ghanaian association studies (Figure 3A).

**Table 4:** Pairwise genetic distances between 6 populations compared.

Group	YRI*	Danes	CEU*	CHB*	JPT*
**Ghanaian**	0.0060	0.2900	0.3520	0.2600	0.3330
**YRI** *		0.2330	0.2940	0.2040	0.2750
**Danes**			0.0060	0.0020	0.0030
**CEU** *				0.0140	0.0010
**CHB** *					0.0090

F_ST_ distance (Latter *et al*., 1972).

*Allele frequency data for the c.371G>C polymorphism for these populations were retrieved from the International HapMap Project database (http://hapmap.ncbi.nlm.nih.gov/cgi-perl/snp_details_phase3?name=rs2073342&source=hapmap28_B36&tmpl=snp_details_phase3). YRI: Yoruba in Ibadan, Nigeria; CEU: (Utah residents with northern and western European ancestry from the Centre d'Etude du Polymorphisme Humain [CEPH]); CHB: Han Chinese from Beijing, China and JPT: Japanese.

### The c.371G>C genotype is subject to selection pressure

**Figure 4:**
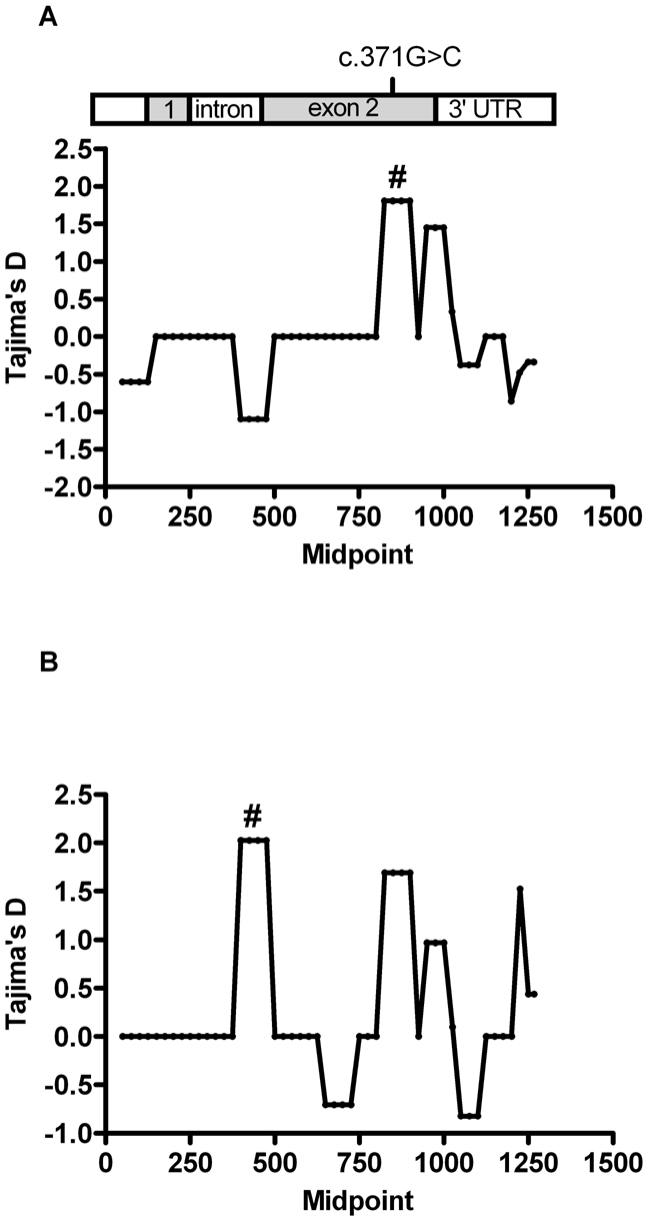
Tajima's D index . Sliding window analysis of Tajima's D index across the Ghanaian (A) and Danish (B) *RNASE3* sequences. Bar at the top shows the sequence region analyzed (nucleotides 1–1183) and the positions of the SNP. Window sizes used were 100 bp with a 25-bp step size. Windows that gave a significant D value *p*<0.10), are indicated above the relevant window midpoint by a hash (#).

To investigate the selective pressures operating on the *RNASE3*, we performed a sliding-window analysis of Tajima's D values across the aligned *RNASE3* sequences for Ghanaians and Danes respectively (Figure 4). This analysis identified a region in *RNASE3* (nucleotides 776–950) that exhibited a nearly significant departure from 0 in the Ghanaians population (D = 1.80; *p*<0.1), indicating that frequency-dependent selection was operating at the c.371G>C SNP.

## Discussion

The findings reported here, together with the observation that levels of ECP increase during acute malaria [20] strongly suggest a role for eosinophils in CM pathogenesis.

Firstly, we have shown that the cytotoxic G-allele of the previously described functional (c.371G>C) polymorphism (rs2073342) of *RNASE3* predisposes to CM in Ghanaian children and we have identified a risk associated haplotype, GGA including this G-allele together with the G-allele of rs2233860 which is associated with increased levels of soluble ECP, and the A-allele of rs8019343. Secondly, the Tajima's D test showed a positive departure from neutrality in the *RNASE3* region encompassing the functional c.371G>C polymorphism. Frequency dependent selection may be a plausible explanation for this phenomenon. Mutations that arise in the population and have the potential to eliminate the toxic activity of ECP may have a survival advantage, leading to their increased frequency in the population. Thirdly, the frequency of the G-allele of the (c.371G>C) polymorphism in the African populations (Ghanaians and the YRI) is significantly reduced compared to non-malarial population (Danes and CEU; CHB and JPT). This finding is in line with the evidence of diversifying selection on the c.371G>C polymorphism and suggests that malaria exerts a strong selection pressure on ECP. Fourthly, c.371G>C and the other SNPs forming the risk haplotype are all in Hardy-Weinberg disequilibrium in the African samples whereas they are in Hardy-Weinberg equilibrium in the Danish samples. Uncharacterised SNPs, insertions or deletions near the 3′ end of a primer may often cause failure in allele amplification [37] and can result in genotype frequency deviations from Hardy-Weinberg equilibrium. However, this is an unlikely explanation for the Hardy-Weinberg disequilibrium observed because DNA sequencing of a larger amplicon revealed no uncharacterised SNPs, insertions or deletions in the initial primer binding regions. Taken together, these findings have led us to propose that ECP and eosinophils are involved in CM pathogenesis. Cerebral malaria pathogenesis is still not clearly understood, however, emerging evidence suggests the possibility that IgE-mediated allergic response or atopy may be risk factors. The chromosome 5q31 locus which has been linked to blood *P. falciparum* parasites density [38] also contains the genes encoding the cytokines IL-4, IL-5, and IL-13, and these have been associated with inflammatory diseases including asthma [39]. IL-5 is a potent activator of eosinophils [40] and together with the elevated IgE levels typically associated with CM [41] might amplify eosinophil-mediated cytotoxicity by ECP against both parasite and host cells.

Studies have shown that ECP efficiently kills some parasites including *P. falciparum*, *in vitro* [21], [42], but the toxic properties of ECP may also lead to tissue damage within the host in conditions such as asthma [43]. The toxic property of ECP is also well documented on several human cell lines, *in vitro*, including epithelial cells [23], [44]–[45]. Although the mechanisms of its cytotoxicity are not fully understood, it has been shown that the 371CC genotype encodes a non-toxic form of ECP [45]. This has been demonstrated using both native ECP and recombinant ECP expressed in several expression systems [23], [44]–[45]. The most likely explanation for this change in cytotoxic activity could be that the substitution of arginine with threonine creates a potentially new glycosylation site in ECP. Indeed Trulson et al [23] demonstrated that the noncytotoxic rECP^124thr^ regained the cytotoxic activity after deglycosylation. Kurtzhals and colleagues observed that ECP plasma levels increased in acute *P. falciparum* infection particularly in CM patients but peripheral eosinophil counts were curiously low. Consequently, they speculated that the low eosinophil counts in these patients may be due to eosinophil destruction or tissue sequestration rather than a decrease in their production [20]. Here we propose that peripheral eosinophils migrate to the post capillary venules during CM where they adhere to intracellular adhesion molecule (ICAM)-1 or other endothelial cellular adhesion molecules (eCAMs) which become up-regulated during CM [46]. Upon binding to (ICAM)-1 through their Beta2 (CD18) integrins, eosinophils release ECP [47] which increases the expression of (ICAM)-1 as demonstrated in nasal epithelia cells [48] suggesting that it might mediate a positive feed-back mechanism. ECP may also be released from eosinophils through stimulation with platelet activating factor (PAF) [49] which is also a potent stimulator of eosinophil adherence [50]. Platelets have recently been proposed to play a central role in the activation of pathogenic inflammation [51], thus PAF might indirectly mediate inflammation through stimulation of eosinophil adherence to the capillaries and the subsequent release of ECP. Knowing that ECP is cytotoxic and may induce necrosis in human primary epithelial cells, we suggest that the release of ECP, together with pro-inflammatory cytokines, may lead to epithelia damage of the capillaries and possibly vascular leakage. Petechial hemorrhaging into the brain is considered a hallmark of CM [52], although the exact contribution of vascular leakage to CM is not known [53].

As the blood-brain barrier becomes compromised during CM, ECP and other plasma proteins may leak into the interstitial space. ECP has been shown to elicit the Gordon phenomenon when injected intracathecally into rabbits, mainly due to its profound neurotoxic effect on the cerebellum compared to other nervous tissue in animal models [19]. Thus, ECP may account for some of the typical cerebellar symptoms observed in CM. In the present study, 371GG which codes for the more cytotoxic and ancestral form of ECP [54] was significantly associated with both CM and low Blantyre coma score. The Blantyre coma score is defined by; (best motor response score) + (best verbal response score) + (eye movement score) [32]; parameters which are largely under the control of the cerebellum [55], suggesting that lower coma scores which typify poor CM prognosis may be a consequence of cerebellar dysfunction due to ECP neurotoxicity. This implies, both ECP neurotoxicity as well as cytotoxicity are either stronger in the native protein (ECP^124arg^) than in the mutant form ECP^124thr^ or fully lost in the mutant since the later was not associated with CM and was only occasionally associated with lower coma scores in the present study.

In the present study we found a large difference between African and non-African samples as exemplified by the distribution of the c.371G>C genotype. The 371CC genotype was dominant in the Ghanaian samples compared to Danish samples (56% vs. 12%) while the opposite was observed for the 371GG genotype (15% vs 53%). This finding is consistent with data for four populations from the International HapMap Project database and also with other studies where this SNP was quantified in samples from Uganda, Sudan, and Sweden [25]. Likewise, the CM associated GGA haplotype was the most common haplotype found in 69% of the Danish samples, whereas it was only found in 18% of the Ghanaian samples. The risk haplotype GGA implies higher intracellular content of cytotoxic ECP and whereas this may crucial in controlling certain infections such as helminths it may also contribute to severe pathology in *P. falciparum* infections as shown in the present study. The high prevalence of the CM associated allele and haplotype in the European population implies a greater than 3 fold likelihood of a European developing CM compared to a Ghanaian if an infection with *P. falciparum* is not treated timely. Conversely, the CGA haplotype was quite uncommon in the Danish samples, but found among 42% of the Ghanaian samples. The most likely explanation for these differences is natural selection e.g. frequency-dependent selection on the c.371G>C SNP. Eriksen et al hypothesized that the predominance of the 371CC genotype is due to positive selection exerted by *Schistosoma mansoni* [25]. However, mortality due to *S. Mansoni* is usually more common in older individuals whereas mortality due to CM is more common in children than in adults. Thus, *P. falciparum* malaria is a more likely cause for the predominance of the C-allele in African populations than *S. Mansoni*. In fact, as the 371CC genotype codes for the non-cytotoxic form of ECP, malaria-endemic populations may be relatively more susceptible to other parasitic infections such as *S. Mansoni* where cytotoxic ECP play a protective role [25]. Thus, ECP may represent an example where one parasite is selecting for a protein variant which makes the same population more susceptible to other parasites. The relatively small sample size might be a limitation in our study. However, the 371G allele frequencies reported here in the present study agrees well with those reported for other populations from malaria endemic areas of East-Africa [25] and with data from the International HapMap Project database, supporting the notion that malaria exerts a strong selection pressure to eliminate the cytotoxic form of ECP. In conclusion, we have identified an association between the 371G allele of *RNASE3* and susceptibility to CM and a risk associated haplotype GGA defined by the markers: rs2073342 (G-allele), rs2233860 (G-allele) and rs8019343 (A-allele) respectively. We have also shown that the *RNASE3* 371GG genotype is subject to selection pressure and we suggest that the predominance of the 371C allele results from a strong selection pressure by *P. falciparum* malaria in endemic populations. Collectively, our results suggest a hitherto unrecognized role for eosinophils in CM pathogenesis.
